# Perioperative assessment of regional ventilation during changing body positions and ventilation conditions by electrical impedance tomography with increased spatial resolution and signal quality

**DOI:** 10.1186/cc14329

**Published:** 2015-03-16

**Authors:** A März, A Ukere, K Wodack, C Trepte, A Waldmann, S Böhm, D Reuter

**Affiliations:** 1 University Medical Center Hamburg-Eppendor, University Hamburg, Germany; 2 Swisstom AG, Landquart, Switzerland

## Introduction

Electrical impedance tomography (EIT) is a functional imaging technology allowing one to regionally monitor aeration of the lungs. We used EIT with increased signal quality and spatial resolution to describe and quantify the regional changes in aeration caused by body position, both during spontaneous breathing and mechanical ventilation in pulmonary healthy patients undergoing laparoscopic prostatectomy.

## Methods

In 40 patients we performed EIT measurements at five points of time (Table 1) with the Swisstom BB2 prototype. Thirty-two electrodes were used to apply weak alternating currents to the thorax and to measure the resulting voltages, from which tomographic images of the changes in regional impedance caused by ventilation were created. We describe the ventilation distribution using a novel EIT lung function parameter called Silent Spaces that provides information about areas that do not receive much or any air during tidal breathing and are divided into nondependent (NSS) and dependent Silent Spaces (DSS) using a reference line that runs perpendicular to the gravity vector right through the centre of ventilation. NSS and DSS are expressed as a percentage of the total lung area.

**Table 1:** 

	1	2	3	4	5
	(sitting, spontaneous breathing)	(supine, spontaneous breathing)	(ventilated, supine position)	(ventilated, 30° head down position)	(ventilated, supine position)
NSS (%)	5.25 ± 2.9	4.12 ± 1.89§	3.05 ± 1.9§	2.8 ± 2.7§	2.67 ± 1.9§
DSS (%)	0.07 ± 0.3	2.29 ± 2.3§	9.23 ± 6.3§	11.5 ± 8.9§*	8.5 ± 5.8§

## Results

Perioperative changes of NSS and DSS are shown in Table 1 as mean ± SD. Statistically significant differences marked by § when compared with 1 or by * when compared with 3 (*P *< 0.05).

## Conclusion

We describe for the first time the mapping of Silent Spaces during spontaneous breathing and changing ventilation conditions and body positions in patients with healthy lungs using EIT. This mapping of Silent Spaces might prove useful for developing perioperative protective ventilation strategies.

